# Recent advances in genome annotation and synthetic biology for the development of microbial chassis

**DOI:** 10.1186/s43141-023-00598-3

**Published:** 2023-12-01

**Authors:** Saltiel Hamese, Kanganwiro Mugwanda, Mutsa Takundwa, Earl Prinsloo, Deepak B. Thimiri Govinda Raj

**Affiliations:** 1 Synthetic Nanobiotechnology and Biomachines Group, Centre for Synthetic Biology and Precision Medicine, Next Generation Health Cluster, CSIR Pretoria, South Africa; 2 Biotechnology Innovation Centre, Rhodes University, PO Box 94, Makhanda, 6140 South Africa; 3 Department of Microbiology, Stellenbosch University, Private Bag X1, Matieland, 7602 South Africa

**Keywords:** Microbial chassis, Synthetic biology, Genome annotation, Gene essentiality, Genome reduction, Lactic acid bacteria (LAB), Lactococcus lactis

## Abstract

This article provides an overview of microbial host selection, synthetic biology, genome annotation, metabolic modeling, and computational methods for predicting gene essentiality for developing a microbial chassis. This article focuses on lactic acid bacteria (LAB) as a microbial chassis and strategies for genome annotation of the LAB genome. As a case study, *Lactococcus lactis* is chosen based on its well-established therapeutic applications such as probiotics and oral vaccine development. In this article, we have delineated the strategies for genome annotations of lactic acid bacteria. These strategies also provide insights into streamlining genome reduction without compromising the functionality of the chassis and the potential for minimal genome chassis development. These insights underscore the potential for the development of efficient and sustainable synthetic biology systems using streamlined microbial chassis with minimal genomes.

## Background

Synthetic biology, precision medicine, and nanotechnology are the three emerging research areas that can be applied as converging fields across various industrial sectors. Synthetic biology is described as the design of new biological parts and the (re-)design of existing biological systems for functional applications. Some synthetic biology applications include the development of synthetic microbes as chassis for recombinant therapeutic production and vaccine development. Microbial chassis are versatile platforms where various bacteria are engineered with genetic components for specific functionalities and address unmet application needs. Synthetic biology, entailing the design and manipulation of biological systems, assumes paramount importance in bioengineering and in silico biology. Computational tools for predicting essential genes and facilitating genome reduction are crucial, offering advantages such as simplified metabolism, improved production, and ease of manipulation. Genome annotation is discussed, focusing on identifying and labeling functional elements in a genome sequence. The generation of synthetic microbes or otherwise called microbial chassis requires the design of minimal genomes that are facilitated through genome-scale metabolic (GSM) models and are critical for chassis development [70]. Furthermore, genome-scale metabolic (GSM) models play a vital role in understanding metabolic capabilities, resource allocation, and adaptation in microbial chassis.

The advantages of chassis with minimal genome have been reported to reduce organism’s complexity by allowing metabolic modeling and functional predictions with higher agility [38]. Improved genome stability has been demonstrated in genome-reduced *Streptomyces* *chattanoogesis* and *E. coli* strains by deleting biosynthetic clusters and error-prone DNA polymerase [12, 18]. Another major advantage is that microbes with reduced genomes require lower bioenergy and this has been demonstrated with the 6.9% reduction of the genome of *Lactococcus lactis* N8 by deleting prophages and genomic islands, resulting in a shortened generation time by 17% [55]. Other benefits of genome-reduced strains include increased production of desired products, improved transformation efficiency, and ease of genetic manipulation [12]. Finally, genome-reduced strains have the potential to be used for downstream applications such as expressing heterologous genes and producing biomolecules using tailored metabolic pathways [38] due to improved growth characteristics, more straightforward metabolism, and fewer functions being performed within the cell of genome reduced strains. This study outlines computational tools for predicting essential genes and designing genomic deletions to facilitate genome reduction. This study has demonstrated the application of computational synthetic biology using *L.* *lactis* as an example of microbial chassis with potential applications in vaccine development.

### Microbial chassis

Choosing the right microbe as a microbial chassis to re-engineer is critical for synthetic biology-driven applications. Engineering of bacterial chassis is considered the most sought-after versatile platform due to robustness, smaller genome size, and simple transcriptional and translational control. Several microbes like *Mollicutes*, *Pseudomonas*, *Escherichia coli (E. coli)*, *Comamonas testosteroni*, and *Bacillus subtilis (B. subtilis)* have been tailored as microbial chassis. *Mollicutes* chassis which are characterized by their absence of cell walls offer insights into the fundamental boundaries of cell survival and division [23]. *Pseudomonas* chassis excels in metabolizing aromatic compounds, enhancing heterologous gene expression. Large-scale genomic deletions in *Pseudomonas putida* chassis yield cells with robust growth [39, 40]. Similarly, *E. coli* chassis with deleted insertion sequences and auxotrophic phenotypes exhibit improved growth fitness [27]*. Comamonas testosteroni* harnesses its natural pollutant-degrading capabilities, making it a promising bioremediation chassis [1]. *B. subtilis* chassis, including delta6, MG1M, and MGB874, are known for their capacity to enhance extracellular protein productivity. Additionally, gram-positive bacteria, like *B. subtilis*, are favored enzyme producers due to their low immunogenicity and limited extracellular protease production [4, 44, 72]. Furthermore, yeast chassis cells display temperature-sensitive attributes, influencing ethanol and glycerol yields [45]. The choice of microbial chassis depends on specific applications targeted and also requires full genome annotation of the chassis in order to effectively engineer thereby highlighting the significance of host genome annotation.

### Genome annotation

Genome annotation identifies functional elements of a genome sequence, indicating its significance. Annotating a genome entails following these steps: identifying genes (including protein-encoding genes and some RNA-encoding genes), predicting the functions of the identified genes, creating metabolic reconstructions and connecting them to genes, labeling phage insertion sequences and transposons, predicting frameshifts and pseudogenes, and identifying regulatory sites and operons, ultimately creating a list of regulons [51]. Regulons are a group of genes or operons that are upregulated or downregulated as a unit by the same protein in response to the same signal. Several genome annotation tools have been developed. These annotation tools may be automated or manual. Automated gene-annotation tools are often used because of the faster annotation and ease of use. However, it is highly recommended that beginners select automatic and semi-automatic annotation methods [31]. Moreover, automatic annotation algorithms, frequently based on orthologs from distantly related model organisms, cannot yet correctly identify all genes within a genome due to confidence and reliability of outcomes as results from different servers or databases are often dissimilar; obtaining accurate gene sets and model manual annotation is often required [21]. Several pipelines for the annotation of genomes have been developed; examples are in Table 1. The gene or protein sequences identified by structural annotation describing the gene structure (e.g., introns, exons, coding sequences, and start and end coordinates) are linked to biological data in a process known as functional annotation, which usually begins with gene identification or gene calling. The different tools for functional annotation are summarized in Table 2. With many genomes sequenced, computational annotation approaches to characterize genes and proteins from their sequences are essential for designing genome deletions.

**Table 1:** Genome annotation pipelines

Pipeline name	Details	Source	URL
**PGAP**	An automatic prokaryotic genome annotation pipeline, which is a set of computational tools and algorithms that are used to predict the presence and location of genes in the genome of a prokaryotic organism	[63]	https://www.ncbi.nlm.nih.gov/genome/annotation_prok/
**Prokka v1.14.6**	A software tool for rapidly annotating genomes, which uses external feature prediction tools like RNAmmer and Prodigal to identify the coordinates of genomic features within contigs	[61]	https://github.com/tseemann/prokka
**RAST**	A fully automated pipeline for annotating bacterial and archaeal genomes	[10]	https://rast.nmpdr.org/
**MicrobeAnnotator**	A tool that predicts all types of prokaryotic genes from a single or a set of anonymous genomic sequences of varying lengths. This tool is commonly used in the analysis of prokaryotic genomes	[58]	https://github.com/cruizperez/MicrobeAnnotator
**EggNOG-mapper v2.1.9**	A tool for automatically annotating the function of genes based on precomputed orthology assignments	[13]	http://eggnog-mapper.embl.de/
**DRAM**	A tool that uses databases like KEGG and PFAM to organize microbial genomic information into a catalog of microbial traits	[62]	https://github.com/WrightonLabCSU/DRAM
**DFAST**	A pipeline that supports genome submission to the public database DNA Data Bank of Japan (DDBJ) using the GHOSTX algorithm	[67]	https://dfast.ddbj.nig.ac.jp/
**GenSAS**	An online pipeline that provides structural and functional annotations of genomic sequences	[28]	https://www.gensas.org/
**BlastKOALA**	A tool that carries out automated annotation of fully sequenced genomes by using KEGG's internal annotation tool, KOALA, to assign K numbers to KEGG genes using SSEARCH computation	[33]	https://www.kegg.jp/blastkoala/

**Table 2:** Functional annotation tools that can be used in microbial genome annotation

Tool	Description	Source
(Meta)GeneMark	A webpage that provides access to gene prediction in metagenomes	[24]
(Meta)Prodigal	Gene prediction software that is used to identify protein-coding genes in prokaryotic genomes	[29]
MetaGeneAnnotator	Predicts all kinds of prokaryotic genes from a single or a set of anonymous genomic sequences having a variety of lengths	[47]
CDD	Comprises protein domains conserved throughout molecular evolution	[41]
ChEBI	The CHEBI database contains manually curated data on chemical entities	[26]
GO FEAT	A web-based functional annotation tool for genomic and transcriptomic data	[5]
GO	The Gene Ontology database provides a standardized vocabulary for describing gene function	[2, 9]
KEGG	Links genomic data and higher-order functional information	[32]
Rhea	Rhea is a database that contains functional annotations for enzymes and descriptions of metabolic pathways. The annotations in Rhea are based on expert-curated, non-redundant information on biochemical reactions	[43]
InterPro	InterPro provides functional analysis of proteins by classifying them into families and predicting domains and important sites	[53]
NCBI Blast	BLAST is a program that identifies regions of similarity between sequences	[30]
SEED	The SEED database provides genome annotations across thousands of genomes	[52]
UniProt	A freely accessible resource on protein sequences and functional annotation	[11]
RefSeq	RefSeq provides a well-annotated set of sequences, including genomic DNA, transcripts, and proteins	[48]
FunMappOne	FunMappOne is a tool for functional analysis and visualization of gene lists	[60]
g:Profiler	Web server for functional enrichment analysis and additional information mining	[37]
GAEV	A tool that was developed for the construction of complete sets of molecular pathways for non-model organisms using KEGG gene function annotations	[73]
GOPlot	GOPlot is an R package for the functional analysis of gene lists	[69]

### Metabolic modeling

**Fig. 1:**
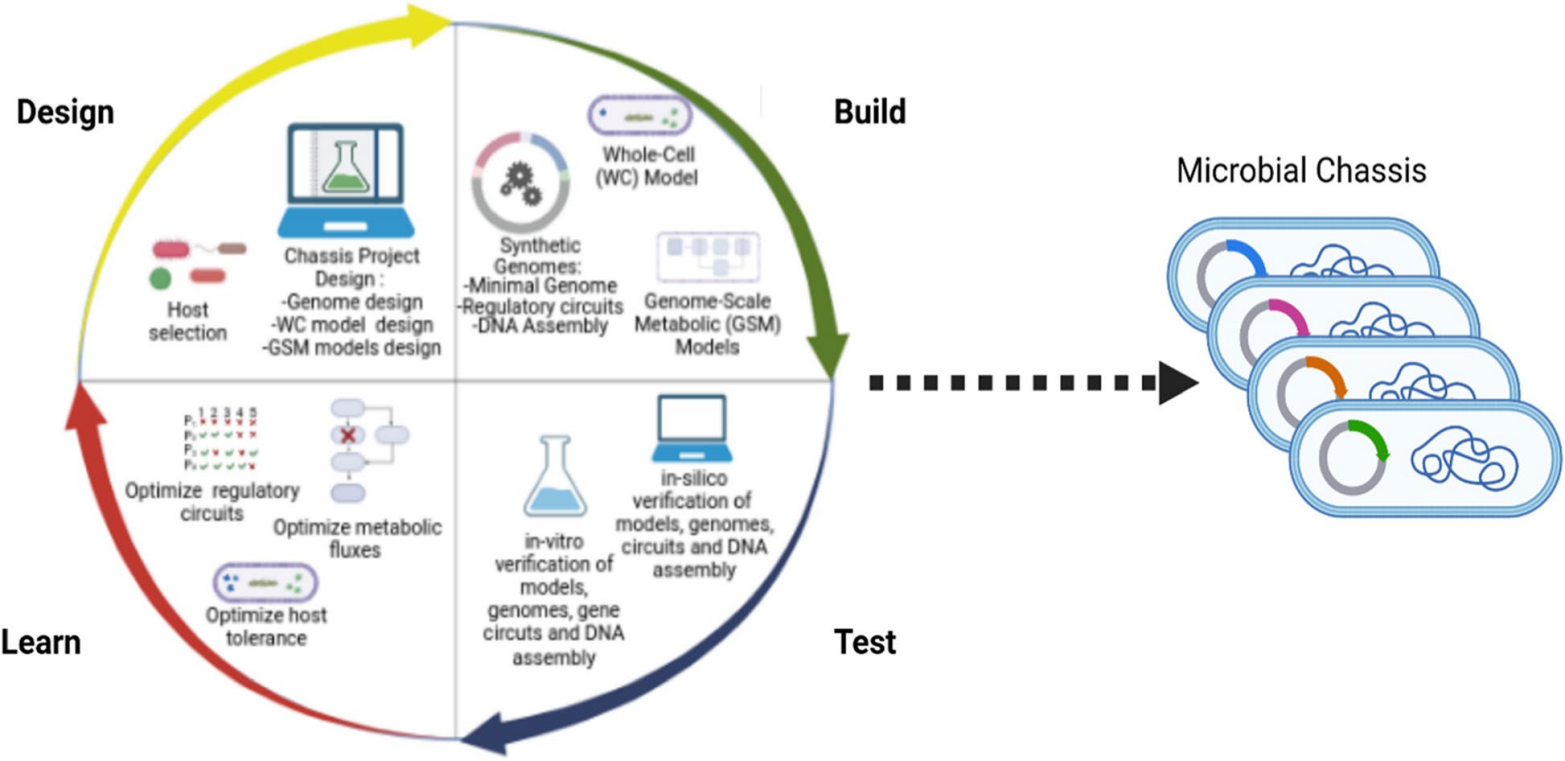
Illustration of the model-guided approach for designing microbial chassis integrated into the synthetic biology design-build-test-learn (DBTL) cycle. This approach requires and utilizes metabolic models, and a minimal synthetic genome to develop a microbial chassis. Illustration created with BioRender

The development of microbial chassis, mainly focusing on LAB (lactic acid bacteria), is significantly propelled by genome-scale metabolic (GSM) models and system biology methodologies. GSM models employ constraints-based modeling, a widely adopted computational method, to map the metabolic pathways and predict phenotypic behavior. Initially applied in the food industry to enhance target product production, GSM models have expanded their utility to system-wide therapeutic targeting for infectious microorganisms and malignancies [3, 15]. Recent advancements, exemplified by creating the iCN1361 GSM model for *Cupriavidus necator* *H16*, demonstrate the integration of omics data and network visualization to improve model applications [54]. Evaluating how well GSM models predict metabolic phenotypes involves contrasting model results with experimental data and subjecting models to in silico simulations under various growth conditions [42]. These GSM models are crucial in understanding a microbial chassis’s metabolic capabilities, predicting metabolic fluxes, and providing insights into resource allocations and adaptation to changing conditions [59]. Moreover, in genome reduction efforts, the models may serve as input alongside essentiality and gene location data [70]. Finally, Fig. 1 illustrates the model-guided approach for designing microbial chassis integrated into the synthetic biology Design-Build-Test-Learn (DBTL) cycle. This approach utilizes metabolic models and a minimal synthetic genome to develop a microbial chassis.

#### Lactic acid bacteria (LAB) as a chosen chassis

Lactic acid bacteria (LAB) have been investigated for their potential use in vaccine development due to their ability to induce a strong immune response. For example, *Lactococcus lactis*, has been modified to deliver antigens and stimulate an immune response in animal models. A recent study explored the expression and secretion of human interleukin-22 (hIL-22) by *Lactobacillus* *reuteri (L. reuteri)*. The results showed that hIL-22 expression and secretion resulted in a growth defect in *L.* *reuteri* and cleavage of most of the secreted hIL-22, although the reason for this is unclear. The study found that changing the signal peptide improved hIL-22 secretion and showed promise for the active hIL-22 on the human intestinal epithelium in vivo, as it was able to stimulate the production of the antimicrobial peptide Reg3α in human intestinal enteroids. LAB have the potential as a vaccine delivery vehicle due to their ability to induce a strong immune response [50]. Synthetic biology tools can be utilized to enhance the properties of LAB for vaccine use, but challenges such as antigen stability and elicitation of an unwarranted immune response must be addressed. The recent study of hIL-22 expression and secretion by *L.* *reuteri* showed promising results, but further research is needed to fully understand the implications and potential limitations.

### Workflow for the design to reduce microbial genome as a chassis

#### Step 1: Choosing lactic acid bacteria (LAB) as host chassis

Lactic acid bacteria (LAB), including genera like *Bifidobacterium*, *Lactobacillus*, *Lactococcus, Leuconostoc*, and *Streptococcus*, play a crucial role as microbial chassis hosts. Lactic acid bacteria (LAB) are considered safe and versatile microbial chassis hosts and are widely used in ingredient production. In recent years, LAB have gained prominence as live delivery vehicles for therapeutic agents, including vaccines, cytokines, enzymes, and allergens. They possess unique attributes such as safety, non-colonizing behavior, and easy elimination from the human body, making them valuable in therapeutic applications [22]. LAB’s potential in vaccine development is notable, given their ability to induce a robust immune response. Synthetic biology tools optimize LAB’s ability to produce, deliver, and express antigens, enhancing their potential as vaccine vectors. However, antigen stability and immune response elicitation must be addressed [50, 57]. Their safety profile, versatility, and potential for immune response induction make them invaluable in developing therapeutic agents and vaccine delivery systems.

#### Step 2: Testing the fitness of Lactococcus lactis as hosts

Lactococcus* lactis* is a mesophilic, Gram-positive, non-motile, non-spore-forming, facultative anaerobe, previously *Streptococcus* *lactis*. It has been used for centuries in producing fermented food products, including cheese and yogurt. It is considered heterofermentative because it produces (S)-lactate as its primary fermentation product and contains genes for enzyme 6-phosphofructokinase (pfkA and pfkB). However, it can have heterofermentative metabolism due to its ability to produce diacetyl, (S)-acetoin, and acetaldehyde, as well as (S)-lactate. Such characteristics made *L.* *lactis* a microorganism of industrial importance. Metabolic efforts of this bacterium have also led to the production of B vitamins (folate and riboflavin), biofuels (ethanol), and therapeutics [65]. Due to its industrial importance, *L.* *lactis* has been categorized as GRAS (generally recognized as safe) by the Food and Drug Administration (FDA).

#### ***Step 3: ***Predicting*** gene essentiality***

Gene essentiality studies are often performed to determine which genes are essential before reducing an organism’s genome. Previous gene essentiality studies involved comparative genomics in search of homologs and paralogs among closely related species [46]⁠ or systemic inactivation of single individual genes [8, 36]⁠. Experimentally or computationally determined essential gene sets may be deposited into available databases of essential genomic regions. Experimentally determined essential gene sets may be deposited into the following databases: DEG (Database of Essential Genes) 15, OGEE (Online GEne Essentiality), and EGGS (Essential Genes on Genome-Scale) whereas pDEG, NetGenes, and ePath are predicted essential gene set databases. The advantages of incorporating computational tools to predict essential genes include low cost and time efficiency. A few algorithms (a series of steps that attempt to solve a problem) have been developed to identify those regions in the genome that may be eliminated. Algorithms that have been developed to identify essential genes include DELEAT (DELetion design by Essentiality Analysis Tool) and Geptop 2.0 [64, 71]. Geptop 2.0 is simple to use, with an interface to input DNA or protein sequences and receive the predicted essentiality with probabilities of genes or proteins. However, it can only be used with fully sequenced organisms. Essential gene databases and computational programs will continue to be utilized to predict essential genes, facilitating the design of genomic deletions [6, 7, 14, 17, 32, 34, 66].

#### Step 4: Performing enrichment analysis

Once potential genes of interest, including gene essentiality predictions, are identified through a large-scale screening, the subsequent challenge is discerning false positives and negatives within these predictions. Integrating gene annotations with the genes of interest is vital to uncovering and evaluating enriched functions of interest. Gene set enrichment analysis is a valuable method for identifying functional classes overrepresented within sets of genes or proteins. Tools such as STRING-db [66] and FUNAGE-Pro [19] play crucial roles in annotating biological functions from gene sets generated through analyses of differential gene or protein expression. The primary data sources for these tools are the complete bacterial genomes housed in the NCBI RefSeq and Genbank databases [16]. The identified protein sequences are mapped against the reviewed and manually curated prokaryote database embedded in UniProt [11]. Functional classes like GO, KEGG, InterPro, and COG can be assigned to each protein, utilizing the UniProt protein annotation. The statistical method for the gene set enrichment analysis is “hypergeometric testing,” employed to identify overrepresented class IDs [20]. This statistical test relies on four key parameters: population size (total annotated genes in the genome), population identified as successful (genes with significant differential expression), sample size (genes in a class-ID), and sample identified as successful (significant values in the class-ID). Additionally, we apply a Benjamini–Hochberg multiple-testing correction to compute the final *P* value, which facilitates the development of ranking scores for visualization purposes, revealing enrichment patterns within the gene sets under investigation.

#### Step 5: Computational design of genome reduction

As more is learned about bacterial genomes, deciding which genes to remove and how to remove those genes becomes increasingly complex. A few computational programs have been developed to assist in the deletion selection and genome design. Moreover, there needs to be more ability to analyze and evaluate genomic designs and an overwhelming number of genome configurations, even for bacteria with small genomes. In genome minimization, two main approaches are used: the top-down approach and the bottom-up approach. The top-down approach involves deleting non-essential genomic regions from an existing genome until the reduced genome supports desired growth yield and rate [68, 70].

**Fig. 2:**
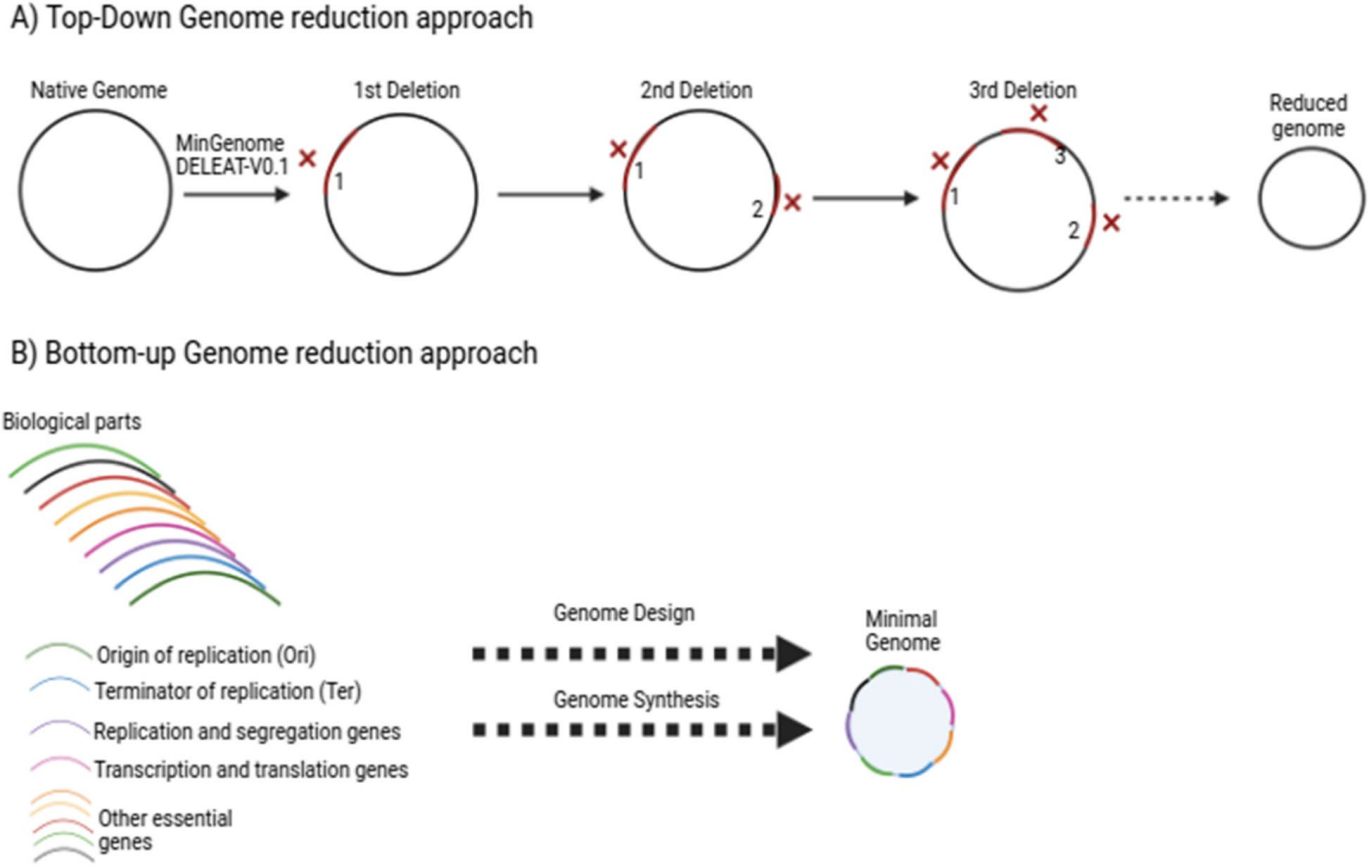
Illustration of the two different genome minimization strategies. **A** The top-down genome minimization approach. DELEAT-v0.1 and MinGenome are examples of tools to design minimal genomes using the top-down strategy. **B** The bottom-up genome minimization approach, where well-characterized, reliable, and context-independent biological parts are constructed into a minimal genome

On the other hand, the bottom-up approach entails designing and building an artificially synthesized genome from scratch using enzymatic assembly [25],K. [35]. Moreover, Fig. 2 compares the two approaches. The top-down approach is primarily used compared to the bottom-up approach due to the cheaper cost and relative ease of the underlying procedures associated with the top-down genome reduction strategy (K. [35]. Both approaches are essential for advancing our understanding of the genetic basis of life and for developing efficient and sustainable biotechnological systems such as microbial chassis.

#### Step 6: Gene circuit design

The availability of gene essentiality data makes it plausible to achieve genome minimization using the bottom-up or top-down approaches⁠. In addition to making gene essentiality predictions, MinGenome and DELEAT computer programs may further be utilized for the in silico top-down reduction of bacterial genomes, with the ability to design large genomic deletions to minimize the organism’s genome [64, 70]. In chassis development, gene circuits are pivotal in controlling gene expression levels and implementing feedback mechanisms to enhance yields and optimize cell populations. The construction of genetic circuits involves assembling well-characterized biological parts essential for achieving the desired expression levels within a cellular chassis. Fundamental biological parts used in genetic circuit design include transcriptional switches, functional non-coding RNAs like riboswitches, ribozymes, and aptamers, as well as CRISPR-based genetic switches and toggle switches. Promoters, critical in controlling gene expression, can be combined and regulated to create internal logic circuits, enabling the engineering of complex microbial behaviors. Additionally, promoters can be combined with ribosome binding sites (RBS) to fine-tune gene expression levels [49]. Toggle switches, acting as memory devices, determine when the chassis will produce specific molecules, such as therapeutic compounds. Secretion tags are often added to the polypeptide chains to ensure that the therapeutic molecules produced do not harm the producing cells. CRISPR-based switches, which can repress gene expression, have been developed, although they may impact the growth of the microbial chassis [56].

Thus, gene circuit design is a crucial aspect of chassis development, leveraging well-characterized biological parts and sophisticated tools to engineer microbial behavior and optimize gene expression within a biological chassis for various applications.

## Conclusions

Herein, we reviewed the critical role of computational methods in obtaining a genome-reduced bacterial strain, focusing on the versatile and safe microbial chassis hosts, lactic acid bacteria (LAB), particularly *L. lactis*. LAB, due to their safety profile, non-colonizing behavior, and ease of elimination from the human body, are versatile chassis hosts extensively utilized in ingredient production and emerging as live delivery vehicles for therapeutic agents, including vaccines. Computational tools play a pivotal role in predicting gene essentiality, aiding in the design of a streamlined genome. Machine learning techniques, particularly deep neural networks, have shown promise in predicting essential genes, which may guide downstream genome reduction strategies. Furthermore, advancements in gene circuit design and metabolic modeling significantly contribute to the engineering of microbial behavior, optimizing gene expression for diverse applications.

## Data Availability

Not applicable.
